# Empathy in Clinical Practice: How Individual Dispositions, Gender, and Experience Moderate Empathic Concern, Burnout, and Emotional Distress in Physicians

**DOI:** 10.1371/journal.pone.0061526

**Published:** 2013-04-19

**Authors:** Ezequiel Gleichgerrcht, Jean Decety

**Affiliations:** 1 Institute of Cognitive Neurology (INECO), Buenos Aires, Argentina; 2 Institute of Neurosciences, Favaloro University, Buenos Aires, Argentina; 3 Laboratory of Neurosciences, Diego Portales University, Santiago, Chile; 4 Department of Psychology and Department of Psychiatry and Behavioral Neuroscience, The University of Chicago, Chicago, Illinois, United States of America; Ecole Normale Supérieure, France

## Abstract

To better understand clinical empathy and what factors can undermine its experience and outcome in care-giving settings, a large-scale study was conducted with 7,584 board certified practicing physicians. Online validated instruments assessing different aspects of empathy, distress, burnout, altruistic behavior, emotional awareness, and well-being were used. Compassion satisfaction was strongly associated with empathic concern, perspective taking and altruism, while compassion fatigue (burnout and secondary traumatic stress) was more closely related to personal distress and alexithymia. Gender had a highly selective effect on empathic concern, with women displaying higher values, which led to a wide array of negative and devalued feelings. Years of experience did not influence dispositional measures *per se* after controlling for the effect of age and gender. Participants who experienced compassion fatigue with little to no compassion satisfaction showed the highest scores on personal distress and alexithymia as well as the strongest indicators of compassion fatigue. Physicians who have difficulty regulating their negative arousal and describing and identifying emotions seem to be more prone to emotional exhaustion, detachment, and a low sense of accomplishment. On the contrary, the ability to engage in self-other awareness and regulate one’s emotions and the tendency to help others, seem to contribute to the sense of compassion that comes from assisting patients in clinical practice.

## Introduction

Clinical empathy is an essential element of quality care and is associated with improved patient satisfaction, adherence to treatment, and fewer malpractice complaints. It has been suggested that in contrast to models of “detached concern,” physicians who attempt to understand what their patient is feeling and communicate their concern achieve a number of valuable outcomes for their patients and for themselves [1]. Empathy in medicine is challenging though, because doctors are dealing with the most emotionally distressing situations–illness, dying, suffering in every form–and such situations would normally make an empathic person anxious, perhaps too anxious to be helpful [2]. This painful reality may take its toll on these individuals and can lead to compassion fatigue, burn out, professional distress and result in a low sense of accomplishment and severe emotional exhaustion [3].

A recent source of concern was sparked by a cross-sectional survey [4], which reported high prevalence of distress and diminished altruistic attitudes among medical students. Importantly, students who suffered from personal distress were more susceptible to engaging in dishonest clinical behaviors. This study is a clear example of the kind of critical questions that can be raised regarding the relationship between interpersonal sensitivity, empathy, and care-giving behavior and, among other things, suggests that empathy does not come without costs.

While everybody views empathy as central to health care and the patient-physician relationship, there is less agreement on what the construct of empathy means. In social science, this term is applied to various phenomena that cover a broad spectrum. Empathy has been described in various ways, such as feelings of concern for others that create the motivation to help, experiencing emotions that match those of another individual, knowing what another is thinking or feeling, and even as blurring of the line between self and other [5]. In medicine, empathy often describes the ability to understand another’s experience, to communicate and confirm that understanding with the other person, and then to act in a helpful manner [6]. This conceptual diversity explains the difficulties in measuring empathy. In fact, none of the attempts to quantify it with self-reports, peer ratings, or patient ratings have been able to capture the entire range of affective, cognitive, and behavioral components of empathy [7].

In the past decade, progress has been made towards a more comprehensive definition of empathy, drawing from empirical research in various academic and clinical domains. There is now a converging agreement that empathy is not a single ability but a complex socio-emotional competency that encompasses different interacting components [8]–[13]. Empathic arousal, the first element of empathy to appear during ontogeny, refers to the contagious sharing of the affective state of another. Empathic understanding entails the formation of an explicit mental representation of the emotional state of another person. Empathic concern refers to other-oriented emotion felt for someone in need, which produces a motivational state of increasing the other’s welfare. Finally, emotion regulation enables the control of emotion, affect, drive, and motivation. Even though these components are intertwined and not independent of one another, it is helpful to dissociate them, as each contributes to various aspects of the experience of empathy [9], [14].

Furthermore, mounting evidence from developmental science, affective social neuroscience in normal individuals as well as in individuals with psychopathologies, and lesion studies in neurological patients indicates that empathy and its component processes are underpinned by specific neural systems. These systems are not limited to the cortex, but are primarily associated with the brainstem, subcortical nuclei, autonomic nervous system, hypothalamic-pituitary-adrenal axis, and endocrine systems that regulate bodily states, emotion, and reactivity [15].

Empirical research examining the components of clinical empathy is imperative in order to understand why some studies have shown that empathy declines during medical school and residency [16], [17]. Another important reason for conducting research on the underlying factors that contribute to clinical empathy and potential associated emotional dysfunctions among health-care professionals is the development of educational intervention for medical students [13], [18].

The goal of the current study sought to determine which aspects of empathy are associated with positive or negative outcome (well-being and burnout respectively) in practicing physicians. Validated self-report instruments were used to assess emotional awareness, empathic concern, personal distress, prosocial behavior, burnout, and well-being.

## Methods

The Ethics Committee at the Institute of Cognitive Neurology (INECO, Buenos Aires, Argentina) approved the present study.

### Participants

Potential participants were board-certified physicians who accessed *Intramed* (www.intramed.net), an online portal exclusively for health professionals. A banner was placed in each user’s main page, inviting visitors to voluntarily access an online survey. All participants gave their informed consent by pressing an “I agree” button placed beneath an explanatory letter. Potential respondents were informed of the anonymity of their responses, which was achieved by deleting their names from the database. The banner was visible for a period of 3 months, during which 7,584 respondents completed the survey. All measures were in Spanish.

### Procedure

Participants provided demographic and professional data and responded to a series of questionnaires. All measures included in this study are described below.

#### Demographic and professional background

Participants provided information about (a) age, (b) gender, (c) current work status, (d) field of practice, (e) number of years in the field, (f) number of years in their current workplace, (g) whether they do “on call” shifts, (h) whether they felt valued by their patients, patients’ caregivers, supervisors, colleagues (Y/N for each one), and (i) whether they felt their medical practice had affected their personal lives, (j) number of sick days they had used during the past 12 months, and (k) whether they had taken a leave of absence during the last year.

#### Empathy

Participants completed the Interpersonal Reactivity Inventory (IRI) [19]. Measures derived from the IRI were three 7-item subscales, assessing specific aspects of empathy, namely: Empathic Concern (EC; the tendency to experience feelings of warmth, compassion, and concern for other people), Personal Distress (PD; one’s own feelings of personal unease and discomfort in reaction to the emotions of others), and Perspective Taking (PT; the tendency to adopt the point of view of other people). EC and PD are considered two independent measures of emotional empathy focusing on the self- and other- oriented set of feelings elicited by an agent. PT, instead, is a measure of the cognitive aspect of empathy.

#### Professional quality of life

The Professional Quality of Life Scale V (ProQOL) developed by Stamm [20] was administered, which presents 30 items rated on a 1 (never) to 5 (very often) scale to obtain three measures: (a) compassion satisfaction (CS), or the pleasure derived from being able to do performing one’s job well; (b) burnout (BO), one of the elements of compassion fatigue, particularly associated with feelings of hopelessness and difficulties in dealing with work or in performing one’s job effectively; and (c) Secondary Traumatic Stress (STS), which is another component of compassion fatigue, consisting of work-related secondary exposure to extreme or traumatic stressful events. BO and STS are two aspects of compassion fatigue. The ProQoL thus reflects on positive (CS) and negative (BO, STS) aspects of medical practice. Participants were classified into low, average, and high groups for each subdomain based on the cut-off scores established in the original ProQoL [20], as follows: lo-CS ≤44; 44< avg-CS <57; hi-CS ≥57; lo-BO ≤43; 43< avg-CS <56; hi-CS ≥56; lo-STS ≤42; 42< avg-CS <56; hi-CS ≥56.

#### Alexithymia

In order to assess difficulties with emotional processing and emotional awareness, the 20-item Toronto Alexithymia Scale (TAS) [21] was used. Participants rated personal statements on a 1 (strongly disagree) to 5 (strongly disagree) Likert-scale, resulting in three independent scores: Difficulty Describing Feelings (DDF), Difficulty Identifying Feelings (DIF), and Externally-Oriented Thinking (EOT), which measures the tendency of individuals to focus their attention externally, a cognitive style that avoids introspective thought. A total alexithymia score is calculated as the sum of these three domains. Based on its original instructions, each individual score on the TAS was used to classify participants as not having alexithymia (scores equal to or less than 51), as having borderline alexithymia (scores of 52 to 60), and as having alexithymia (scores equal to or greater than 61).

#### Altruistic behavior

Participants were asked to determine the frequency (“never”, “once”, “at least two or three times”, “at least once a month”) with which they engaged in ten different helping behaviors derived from the Self-Report Altruism scale [22] during the past 12 months. Measures derived from this inventory were an altruistic behavior score (ALT; based on the added frequencies of all 10-items), as well as direct (ALT-D) and indirect (ALT-I) altruism scores based on whether prosocial acts targeted, respectively, a specific (e.g. helping someone with their shopping bags) or an abstract (e.g. donating blood) beneficiary. Each participant’s ALT–D and –I scores were compared individually to determine whether they favored direct or indirect altruistic acts.

Alexithymia and altruistic behavior measures were only made available towards the third half of the response period, resulting in responses obtained for a subset of 1,881 participants. A comparison of key variables between the 5703 physicians reporting the initial set of measures and the 1881 physicians reporting the extended set of questionnaires revealed no significant differences between the samples, therefore validating their equivalency for key variables of the present study (Table S1).

### Statistical Analysis

Comparisons of dependent variables between two groups at a time (e.g. female vs. male participants, more vs. less experienced physicians, etc.) were conducted Student’s *t* test and one-way ANOVA was used to compare variables across three or more groups at a time. As well, ANCOVAs were carried out to control for the effect of potential confounding variables (e.g. age and years of experience when comparing dispositions between male and female physicians). For instances in which the main effect of given factors and their interactions became relevant (e.g. compassion fatigue and compassion satisfaction), a factorial 2×2 ANOVA design was employed. When analyzing categorical variables (e.g. gender), the Fisher exact probability test for contingency tables was used. Correlations between variables were analyzed using Pearson’s correlation coeffcient. The α value for all statistical tests was set at 0.05, two-tailed, but, as expected given the large sample size of participants in the study, most measures rendered significant differences. Differences are shown as Cohen’s *d* scores because the size of the effect when comparing groups is more important than the degree of significance *per se*. Throughout the results section, whenever it is reported one group scored higher or lower than another, the difference was significant at least at p<.01. The methodology follows Cohen‘s [23] classification of effect sizes as small/slightly (*d* = .10 to.39), medium/moderately (*d* = .40 to.69), or large/substantially (*d* >.70). Effect sizes for analyses of categorical data are expressed in terms of Cramer’s V score.

## Results

### Demographic Profile and Professional Background

Participants were 53.5% male (*n* = 4057) and 44.6 (*SD* = 12.1) years old on average and resided in more than 20 different countries (Table S2). They practiced medicine in a wide variety of fields, with 17.8 (*SD* = 11.9) years of medical experience on average. At time of assessment, they had spent an average of 11.9 (SD = 10.8) years working at their current workplaces, while 87 (1.1%) were currently unemployed. Of the 7,497 working physicians, 730 (9.7%) were residents and 46.1% did on-call shifts at least once a month. During the last 12 months, participants had used 8.4 (SD = 33.2) sick days on average and 22.5% had taken a leave of absence for health-related reasons.

### Dispositional Measures

#### Empathy

Participants scored, on average, 31.1 (*SD* = 5.2) on EC, 13.0 (*SD* = 4.5) on PD, and 23.6 (*SD* = 4.8) on PT.

#### Professional quality of life

Scores across the three domains of the ProQoL questionnaire are presented in Table 1.

**Table 1:** ProQoL scores.

	Compassion Satisfaction	Burnout	Secondary Traumatic Stress	
Score	49.5 (10.1)	50.3 (10.2)	50.2 (10.1)	
% low	27.3	26.1	23.5	
% average	47.8	42.6	49.2	
% high	24.9	24.9	27.2	

Mean (*SD*) scores across the different domains of the ProQoL questionnaire. Percentages represent the proportion of participants classified into low, average, and high groups for each subdomain based on the cut-off scores proposed by the original manual.

#### Alexithymia

Participants scored, on average, 10.8 (SD = 4.2) on their DDF, 13.5 (SD = 6.2) on their DIF, and 16.6 (SD = 4.8) on EOT. Average total alexithymia scores were 40.9 (SD = 12.3). Based on each participant’s individual alexithymia score, 80.5% were non-alexithymic, 11.5% had borderline alexithymia, and 8% had alexithymia.

#### Altruistic behavior

Participants scored, on average, 22.0 (SD = 5.1) on the helping behavior inventory based on the frequency of their prosocial acts. The average ALT-D was 2.3 (SD = 0.6) and ALT-I was 2.0 (SD = 0.6). Based on their altruistic acts, it was observed that 67% of participants favored direct prosocial behavior and 33% favored indirect prosocial behavior.

### The Effect of Gender

Men were significantly older (Cohen’s *d* = .47) and had significantly more professional experience (Cohen’s *d* = .44) than women (Table 2). In comparing alexithymia scores, men showed a significantly stronger tendency to focus their attention externally rather than in their own thoughts and feelings (Cohen’s *d* = .29) but were comparable to women in other aspects (DDF: Cohen’s *d* = .11; DIF: Cohen’s *d* = .02). Men and women were also comparable in their altruistic behavior (Cohen’s *d* = .08) both for direct (Cohen’s *d* = .11) and indirect (Cohen’s *d* = .02) acts.

**Table 2:** Comparison of age, years of experience, alexithymia and altruism between men and women.

		Female *n* = 3527/902		Male *n* = 4057/979					
		*Mean*	*SD*	*Mean*	*SD*	*t*	*df*	*p*	*Cohen’s d*
Age		41.6	11.3	47.2	12.1	20.5	7582	<.001	.47
Experience (years)		15.1	10.8	20.2	12.3	19.2	7582	<.001	.44
Alexithymia	DDF	10.51	4.23	11	4.33	2.52	1879	.01	.11
	DIF	13.42	5.99	13.57	6.31	0.52	1879	.60	.02
	EOT	15.92	4.76	17.31	4.83	6.26	1879	<.001	.29
	TOT	39.84	12.1	41.88	12.35	3.60	1879	<.001	.17
Altruism	ALT	22.2	4.89	21.81	5.33	1.67	1879	.10	.08
	ALT-D	2.30	0.55	2.24	0.59	2.33	1879	.02	.11
	ALT-I	2.02	0.62	2.04	0.64	0.34	1879	.65	.02

DDF = Difficulty Describing Feelings, DIF = Difficulty Identifying Feelings, EOT = Externally-Oriented Thinking, ALT-D = Direct Contact Altruism, ALT-I = Indirect Contact Altruism.

**Figure 1:**
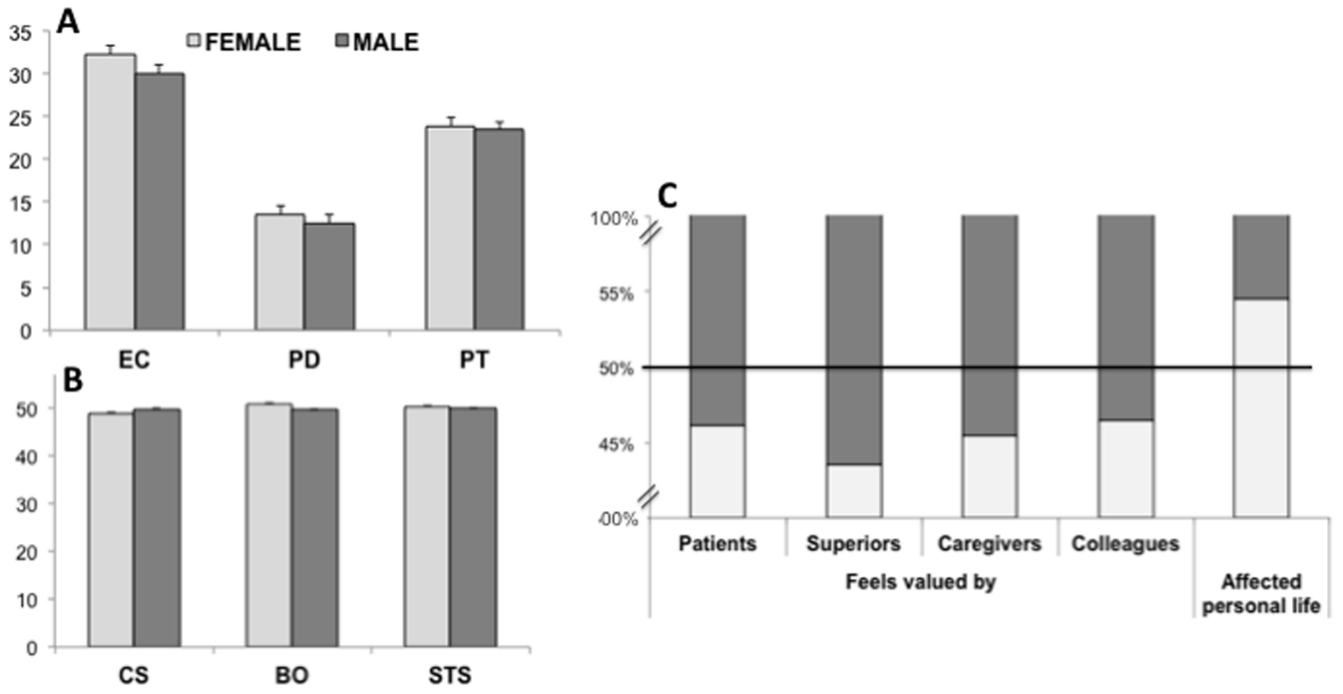
Comparison of (A) empathy subscores, (B) professional quality of life subscores, and (C) work-related personal perceptions between male and female participants. Error bars in A and B represent *S. E. M.*

As shown in Figure 1, women reported significantly higher values in all components of empathy, but the effect was especially robust for EC (*t*_7582_ = 18.4, *p*<.001, Cohen’s *d* = .42), followed by PD (*t*_7582_ = 9.39, *p*<.001, Cohen’s *d* = .22) and almost negligible for PT (*t*_7582_ = 3.32, *p*<.001, Cohen’s *d* = .08). Significant differences found on CS (*t*_7582_ = 3.83, *p*<.001) and BO (*t*_7582_ = 5.71, *p*<.001) had small effect sizes (Cohen’s *d* = .09 and.13, respectively) and STS did not differ between men and women (*t*_7582_ = 1.72, *p* = .08, Cohen’s *d* = .04). Covarying for age and years of experience further strengthened the results found both for empathy (EC: *F*_1,7580_ = 387.9, *p*<.001, Cohen’s d = .45; PD: *F*_1,7580_ = 97.2, *p*<.001, Cohen’s d = .23; PT: *F*_1,7580_ = 15.6, *p*<.001, Cohen’s d = .09) and professional quality of life (CS: *F*_1,7580_ = 9.26, *p*<.01, Cohen’s d = .06; BO: *F*_1,7580_ = 14.0, *p*<.001, Cohen’s d = .09; STS: *F*_1,7580_ = 5.45, *p* = .02, Cohen’s d = .06), revealing a highly specific effect of gender on empathic concern but not other aspects of empathy or compassion satisfaction/fatigue.

In addition, compared to male participants, female participants felt less valued by patients (*χ* ^2^ = 7.0, *p*<.01, Cramer’s V = .03) and their caregivers (*χ* ^2^ = 24.4, *p*<.001, Cramer’s V = .06), and by their colleagues (*χ* ^2^ = 39.6, *p*<.001, Cramer’s V = .07) and superiors (*χ* ^2^ = 51.2, *p*<.001, Cramer’s V = .08). More women than men (*χ* ^2^ = 154.2, *p*<.001, Cramer’s V = .15) reported feeling that their jobs had affected their personal lives negatively (Figure 1C).

### The Effect of Professional Experience

Participants were divided into those who had more and less experience based on whether their individual years of medical practice were above or below the sample’s mean. Significant differences between more and less experienced physicians on empathy were found exclusively on EC (*t*_7582_ = 2.67, *p*<.01), although with a limited effect size (Cohen’s *d* = .06). With regards to professional quality of life, slight significant differences were found on all domains (CS: *t*_7582_ = 3.37, *p* = .001, Cohen’s *d* = .08; BO: *t*_7582_ = 6.78, *p*<.001, Cohen’s *d* = .16; STS: *t*_7582_ = 2.91, *p*<.01, Cohen’s *d* = .07). These very small effect size differences remained when we compared participants in the extreme percentiles of years of experience (Table S3).

Similarly, significant differences were found on the TAS-20 total score (*t*_1879_ = 2.67, *p*<.01) and the direct (*t*_1879_ = 2.96, *p*<.01) and indirect (*t*_1879_ = 5.1, *p*<.001) contact altruistic acts, but the effect sizes were small to moderately small (Cohen’s *d* = 0.12, 0.14, and.23, respectively).

Because a significantly higher (*χ* ^2^ = 203.2, *p*<.001, Cramer’s V = .17) proportion of women (60.1%) than men (43.7%) were in the less experienced group, dispositional measures were compared by covarying for the effect of gender, as well as for the effect of age, which was, as expected, significantly higher (*t*_7582_ = 112.2, *p*<.001, Cohen’s *d* = 2.58) in the more experienced group (54.4±7.5 vs. 35.3±7.2). No significant differences were found between any of the components of empathy, professional quality of life, alexithymia or altruism after controlling for the effect of age and gender, suggesting that the years of experience in clinical practice *per se* do not independently influence dispositional measures (Table 3). Years of experience correlated significantly, but remarkably slightly, with CS (*r* = .03, *p*<.01), BO (*r* = −.09, *p*<.001), STS (*r* = .04, *p* = .001), as well as total alexithymia scores (*r* = −.07, *p*<.01) and its subdomains [DDF (*r* = −.05, *p* = .03), DIF (*r* = −.06, *p* = .01), EOT (*r* = −.06, *p* = .02)]. No significant correlations were found between years of experience and EC (*r* = .02, *p* = .06), PD (*r* <.01, *p* = .58), or PT (*r* = .01, *p* = .23). A significantly inverse correlation was found between years of experience and altruistic behavior (*r* = −.05, *p* = .04), but again, correlation coefficients were extremely small.

**Table 3:** Dispositional measures.

		Less Experienced*n* = 3891/899		More Experienced*n* = 3693/982					
		*Mean*	*SD*	*Mean*	*SD*	*t*	*df*	*p*	*Cohen’s d*
Empathy	EC	30.9	5.4	31.3	5.1	2.67	7582	<.01	.06 | <.01
	PD	13.0	4.5	13.0	4.5	0.48	7582	.63	.01 | <.01
	PT	23.5	4.9	23.7	4.7	1.29	7582	.20	.03 | <.01
Professional Quality of Life	CS	38.7	7.4	39.3	7.0	3.37	7582	.001	.08 | <.01
	BO	22.7	6.8	21.6	7.0	6.78	7582	<.001	.16 | <.01
	STS	16.2	6.8	16.7	7.7	2.91	7582	<.01	.07 | <.01
Alexithymia	DDF	11.0	4.3	10.6	4.3	2.00	1879	.05	.09 | <.01
	DIF	13.8	6.2	13.2	6.1	2.1	1879	.04	.09 |.06
	EOT	16.9	4.9	16.4	4.8	2.4	1879	.2	.11 |.06
	TOT	41.7	12.5	40.2	12.1	2.7	1879	<.01	.12 |.06
Altruism	ALT	22.1	5.0	21.9	5.2	0.45	1879	.65	.02 |.06
	ALT-D	2.3	0.6	2.2	0.6	2.96	1879	<.01	.14 | <.01
	ALT-I	2.0	0.6	2.1	0.6	5.1	1879	<.001	.24 |.13

Comparison of dispositional measures between physicians grouped based on whether their individual experience in the medical field was above (More Experienced) or below (Less Experienced) the sample’s average. Effect sizes are reported both before (left) and after (right) covarying for age and gender.

EC = Empathic Concern, PD = Personal Distress, PT = Perspective Taking, CS = Compassion Satisfaction, BO = Burnout, STS = Secondary Traumatic Stress, DDF = Difficulty Describing Feelings, DIF = Difficulty Identifying Feelings, EOT = Externally-Oriented Thinking, TOT = Total Alexithymia score, ALT = Total Altruism score; ALT-D = Direct Contact Altruism, ALT-I = Indirect Contact Altruism.

### Relationship between Professional Quality of Life and Dispositions

#### Relationship between dispositions

In order to better understand the way in which individual dispositions affect professional quality of life, we initially conducted multiple regression analyses on its positive (CS) and negative (BO, STS) aspects, introducing EC, PD, PT, DDF, DIF, EOT, ALT-D and ALT-I as predictor variables. Total scores (e.g. total TAS or total altruism) were not included in order to avoid the effects of multicollinearity. Table 4 presents multiple regression results for the three domains of professional quality of life, all three of which rendered significant models (all *p*<.001).

**Table 4:** Multiple regression analysis on the positive (CS) and negative (BO, STS) aspects of professional quality of life.

		Compassion Satisfaction	Burnout	Secondary Traumatic Stress
R		.43	.32	.56
Adjusted R square		.18	.10	.31
*F* (8,1872)		51.7	26.3	106.5
*P*		<.001	<.001	<.001
Empathy	EC	*β* = .12, *p*<.001	*β* = .11, *p*<.001	*β* = .13, *p*<.001
	PD	*β* = −.22, *p*<.001	*β* = .14, *p*<.001	*β* = .35, *p*<.001
	PT	*β* = .13, *p*<.001	*β* = −.03, *p* = .28	*β* = −.04, *p* = .10
Alexithymia	DDF	*β* = −.07, *p* = .04	*β* = .04, *p* = .24	*β* = .06, *p* = .03
	DIT	*β* = −.07, *p* = .02	*β* = .18, *p*<.001	*β* = .26, *p*<.001
	EOT	*β* <.01, *p* = .98	*β* = −.04, *p* = .08	*β* = .03, *p* = .23
Altruism	ALT-D	*β* = −.02, *p* = .35	*β* = .03, *p* = .23	*β* = .04, *p* = .07
	ALT-I	*β* = .19, *p*<.001	*β* = .08, *p*<.01	*β* = .04, *p* = .07

Beta values are standardized coefficients.

EC = Empathic Concern, PD = Personal Distress, PT = Perspective Taking, CS = Compassion Satisfaction, BO = Burnout, STS = Secondary Traumatic Stress, DDF = Difficulty Describing Feelings; DIF = Difficulty Identifying Feelings; EOT = Externally-Oriented Thinking; TOT = Total Alexithymia score; ALT = Total Altruism score; ALT-D = Direct Contact Altruism; ALT-I = Indirect Contact Altruism.

EC significantly and similarly predicted CS, BO and STS. PD also significantly predicted all domains of professional quality of life, but in different manners: inversely for CS and proportionally with the negative aspects of professional quality of life (BO and STS). PT was exclusively predictive of the positive aspect (CS), but not predictive of BO or STS. Of the different aspects under the umbrella of alexithymia, EOT was not predictive of any of the variables, while DDF and DIT were predictive of both positive and negative aspects of professional quality of life, with stronger significant values for the latter. Only ALT-I, and not ALT-D, predicted CS (more markedly) and BO.

**Figure 2:**
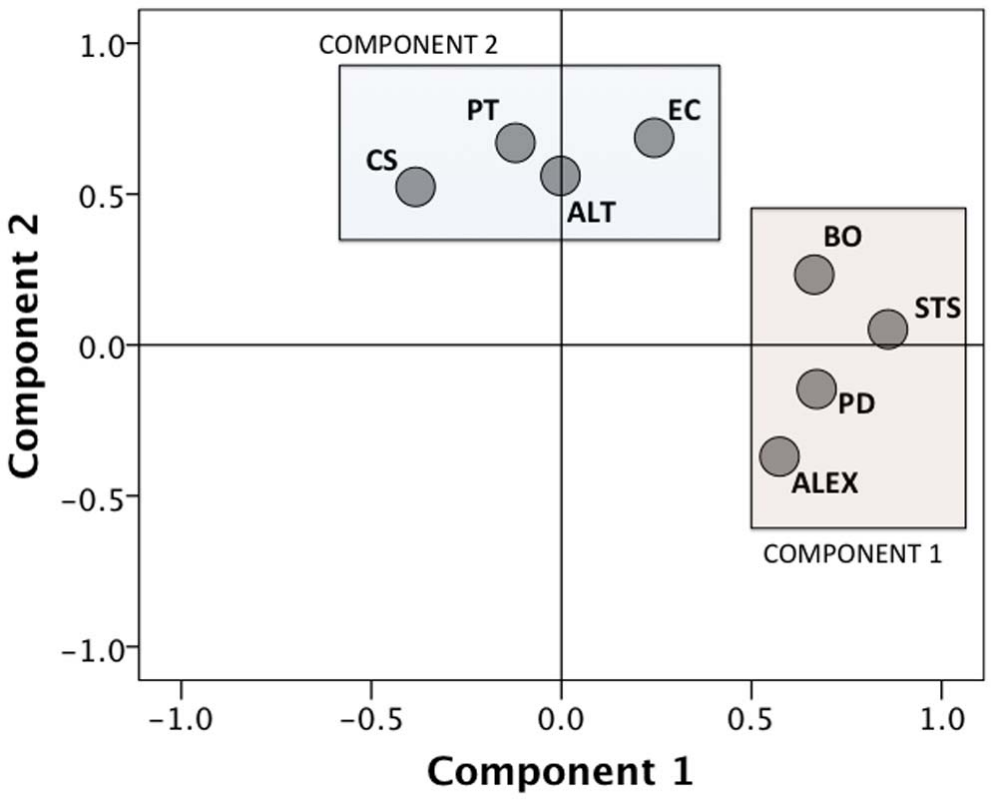
Factor analysis on dispositional measures revealed two principal components.

Principal component analyses (PCA) using varimax rotation (to reduce dimensionality of the data set) were conducted using measures available for the whole sample (PCA1: IRI and ProQoL subscales) and for a smaller subset (PCA2: IRI, ProQoL, TAS-20, ALT subscales) in order to explore the way in which different dispositional measures were related to each other. Only factors with Eigenvalues >1 were extracted, and factor loadings were considered meaningful if r >.50. The appropriateness of our large data set to be subjected to PCA was indicated by the Kaiser-Meyer-Olkin measure of sampling adequacy (PCA1: KMO = .62; PCA2: KMO = .70) and Bartlett’s test of sphericity (PCA1: *χ* ^2^ = 9948.2, df = 15, p<.0001; PCA2: *χ* ^2^ = 4456.4, df = 55, p<.0001). As shown in Table 5, for PCA1, two components were extracted (Eigenvalues of 2.3 and 1.4, respectively), which explained 38.4% and 23.5% of the variance, respectively (cumulative: 61.9%). Component 1 loaded the negative aspects of professional quality of life (BO and STS) together with PD. Component 2 loaded CS together with EC and PT. For PCA2, four components were extracted (Eigenvalues of 2.88, 1.84, 1.22, 1.01, respectively), which explained 26.1%, 16.8%, 11.1%, and 9.1% of the variance (cumulative: 63.1%). As shown in Table 6, components 1 and 4 of PCA2 were equivalent to Components 1 and 2 from PCA1, respectively. Component 2 gathered the different aspects of alexithymia and Component 3 clustered both types of altruistic acts. For this reason, PCA was repeated on PCA2 using the total scores of alexithymia and altruism (KMO = .68, *χ* ^2^ = 2229.6, df = 28, p<.0001), further reducing the variables into two components (Eigenvalues of 2.3 and 1.6, respectively). As it is evident from Figure 2, Component 1 loaded PD, BO, STS, and alexithymia, while Component 2 loaded EC, PT, CS and altruism.

**Table 5:** Principal Component Analyses (PCA) including IRI and ProQoL subscales.

		Component	
		1	2
Professional Quality of Life	CS	−.397	**.589**
	BO	**.834**	−.178
	STS	**.841**	.113
Empathy	EC	.191	**.811**
	PD	**.691**	.012
	PT	−.146	**.738**

**Table 6:** Principal Component Analyses (PCA) including IRI, ProQoL, TAS-20, ALT subscales.

		Using subtotal scores				Using total scores	
		1	2	3	4	1	2
Professional Quality of Life	CS	−.455	−.053	.331	**.606**	−.384	**.524**
	BO	**.694**	.051	.211	.073	**.665**	.233
	STS	**.815**	.248	.057	.042	**.859**	.052
Empathy	EC	.214	−.026	.085	**.760**	.244	**.686**
	PD	**.661**	.157	−.219	−.011	**.671**	−.147
	PT	−.120	−.146	.023	**.765**	−.121	**.670**
Alexithymia	DDF	.217	**.866**	−.062	−.021	**.574**	−.370
	DIF	.309	**.827**	−.006	.011		
	EOT	−.052	**.614**	−.086	−.348		
Altruism	ALT-D	.021	−.028	**.769**	.160	−.003	**.560**
	ALT-I	−.003	−.076	**.847**	−.024		

#### Empathy

**Figure 3:**
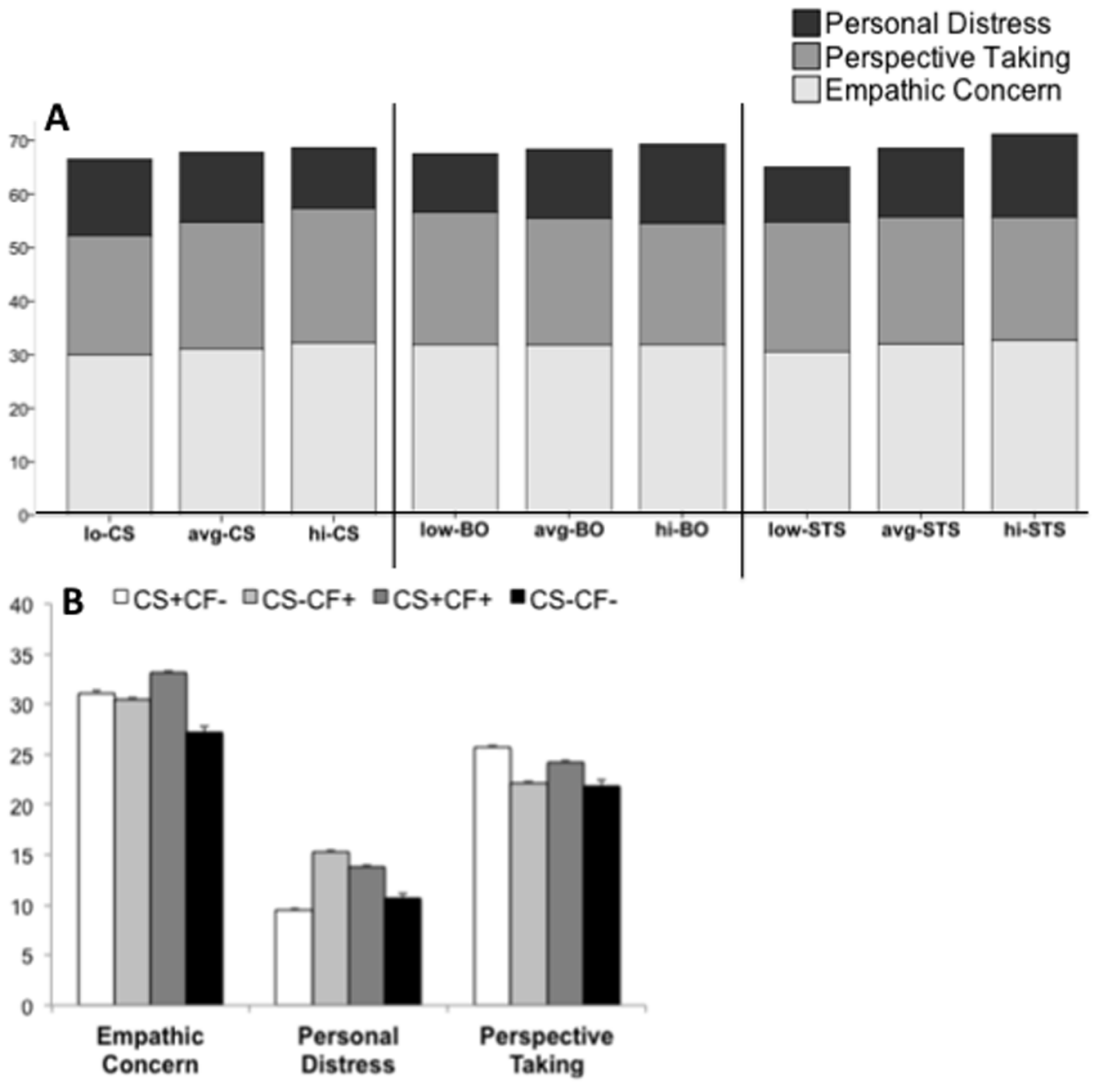
Empathy subdomain scores for participants (A) in the low, average, and high groups across the different aspects of professional quality of life and (B) grouped based on their compassion satisfaction (CS) and compassion fatigue (CF) profiles. Error bars are *S. E. M.*

As revealed by Figure 3A, hi-CS participants had significantly higher scores on EC (*F*_2,7581_ = 178.8, *p*<.001, Cohen’s d = 0.43) and PT (*F*_2,7581_ = 93.3, *p*<.001, Cohen’s d = 0.32) with significantly lower scores on PD (*F*_2,7581_ = 225.5, *p*<.001, Cohen’s d = 0.49), compared with both avg- and lo-CS participants. When comparing different empathy domains across BO groups, significant differences were not found for EC (*F*_2,7581_ = 0.84, *p* = .92), but avg- and hi-STS participants had significantly lower values of PT (*F*_2,7581_ = 102.1, *p*<.001, Cohen’s d = 0.32) and remarkably higher scores of PD (*F*_2,7581_ = 435.8, *p*<.001, Cohen’s d = 0.68). Across the different STS groups, very slight significant differences were found on PT (*F*_2,7581_ = 32.5, *p*<.001, Cohen’s d = 0.19), with lo- and avg-STS participants scoring significantly lower than those in the hi-STS group (*p*<.001). EC was also significantly lower among lo-STS (*F*_2,7581_ = 86.1, *p*<.001, Cohen’s d = 0.30), but the effect was remarkably significant for PD (*F*_2,7581_ = 772.0, *p*<.001, Cohen’s d = 0.91), with hi-STS scoring higher (*p*<.001) than both avg- and lo-STS.

We then sought to explore the relationship between empathy and professional quality of life by studying extreme cases of compassion satisfaction and compassion fatigue (CF).

- CS+CF– : Participants who **exclusively** experience the positive aspects of being compassionate (hi-CS & lo-BO & lo-STS).
- CS–CF+ : Participants who **exclusively** experience the negative aspects of being compassionate (lo-CS & hi-BO/hi-STS).
- CS+CF+ : Participants who experience the positive and negative aspects of being compassionate (hi-CS & hi-BO/hi-STS).
- CS–CF– : Participants who experience neither the positive nor the negative aspects of being compassionate (lo-CS & lo-BO & lo-STS).

Following these criteria, 6.9% were CS+CF– (*n* = 522), 16.9% were CS–CF+ (*n* = 1281), 4.9% were CS+CF+ (*n* = 373), and 1.3% were CS–CF– (*n* = 100). The remaining 70% of the sample included participants whose ProQoL scores were average for one or more domains.

Significant differences across the groups were slight for age (*F*_3,2272_ = 5.9, *p*<.001, Cohen’s *d* = 0.18) and years of education (*F*_3,2272_ = 4.1, *p*<.01, Cohen’s *d* = 0.14). A significantly higher number of participants in the CS–CF+ group (55.3%) reported to working “on call” shifts (*χ* ^2^ = 46.6, df = 3, *p*<.001, Cramer’s V = .15), while the CS+CF– had the least number (38.7%) of participants working these shifts. Remarkably, a main effect of compassion fatigue (*F*_1,2272_ = 5.14, *p* = .02, Cohen’s *d* = 0.15) but not compassion satisfaction (*F*_1,2272_ = 0.45, *p* = .50, Cohen’s *d* <.01) was found for the number of sick days taken by the participants, with CS−CF+ taking the highest number of sick days (11.7±40.6), followed by CS+CF+ (8.4±28.6), almost double the number of sick days taken by CS+CF− (5.4±22.9) and CS−CF− (4.9±9.95) physicians. There was no significant interaction between compassion satisfaction and fatigue for the number of sick days taken (*F*_1,2272_ = 0.82, *p* = .37, Cohen’s *d* <0.01). As many as 68.1% of physicians in the CS−CF+ group took a medical leave of absence at some point during the past 12 months, a proportion which was significantly higher (*χ* ^2^ = 51.4, df = 3, *p*<.001, Cramer’s V = .16) than in the other groups (14.3% CS+CF−, 14.6% CS+CF+, 3% CS−CF−).

For EC, a main effect of compassion satisfaction (*F*_1,2272_ = 106.2, *p*<.001, Cohen’s *d* = 0.43) and a strong main effect of compassion fatigue (*F*_1,2272_ = 64.3, *p*<.001, Cohen’s *d* = 0.34) were found. Physicians who experienced compassion satisfaction exhibited higher EC scores than physicians who did not, and participants who experienced compassion fatigue still showed higher EC scores than physicians who did not. The interaction between CS and CF was not significant for EC (*F*_1,2272_ = 3.41, *p* = .07, Cohen’s *d* = 0.04). For PD, a main effect of compassion satisfaction (*F*_1,2272_ = 26.0, *p*<.001, Cohen’s *d* = 0.21) and a strong main effect of compassion fatigue (*F*_1,2272_ = 271.8, *p*<.001, Cohen’s *d* = 0.70) were found, such that physicians who experienced compassion fatigue always exhibited higher PD scores than physicians who did not, and participants who experienced compassion satisfaction showed lower PD scores than physicians who did not. The interaction between CS and CF was not significant for PD (*F*_1,2272_ = 0.35, *p* = .55, Cohen’s *d* <0.01). Interestingly, one-way ANOVA group post hoc analyses revealed that CS+CF– showed even smaller PD values than CS–CF– (*p*<.05). For PT, a strong main effect of compassion satisfaction (*F*_1,2272_ = 98.2, *p*<.001, Cohen’s *d* = 0.42) and a main effect of compassion fatigue (*F*_1,2272_ = 4.4, *p*<.001, Cohen’s *d* = 0.08) were found, as well as a significant interaction between these factors (*F*_1,2272_ = 9.21, *p*<.01, Cohen’s *d* = 0.13), such that CS+CF– physicians exhibited the highest PT scores and CS–CF– physicians exhibited the lowest scores for this domain. In turn, experiencing compassion fatigue was associated with higher PT scores if also experiencing compassion satisfaction.

#### Alexithymia

**Figure 4:**
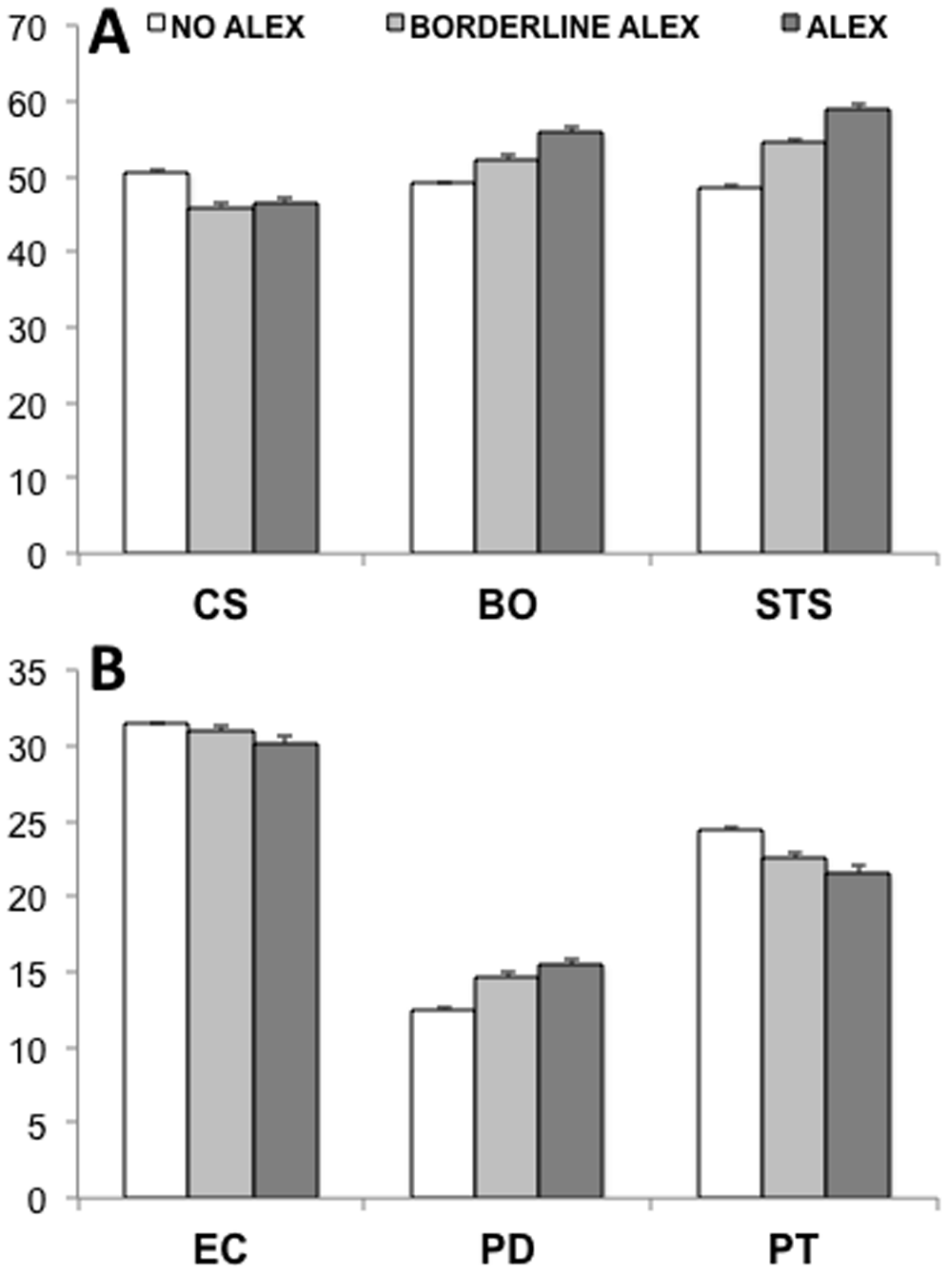
Comparison of (A) professional quality of life and (B) empathy across physicians who had no alexithymia, borderline alexithymia and alexithymia, as determined by their scores on the TAS-20.

When looking at the effect of alexithymia (ALEX) on the different aspects of professional quality of life and empathy, significant differences were found between physicians with ALEX, borderline ALEX, and no ALEX (Figure 4). Medical doctors with no ALEX showed significantly higher (*F*_2,1878_ = 28.7, *p*<.001, Cohen’s *d* = .36) scores than those with ALEX (*p*<.001) and borderline ALEX on CS and significantly lower scores than both groups (*p*<.001) on the negative aspects of professional quality of life, i.e. BO (*F*_2,1878_ = 38.6, *p*<.001, Cohen’s *d* = .40) and STS (*F*_2,1878_ = 109.0, *p*<.001, Cohen’s *d* = .68). While the significant differences found between the groups on EC (*F*_2,1878_ = 4.62, *p* = .01) were slight (Cohen’s *d* = .15), a stronger effect of ALEX was found on PD (*F*_2,1878_ = 36.2, *p*<.001, Cohen’s *d* = .39) and particularly on PT (*F*_2,1878_ = 48.9, *p*<.001, Cohen’s *d* = .46). On the former, physicians with no ALEX showed lower levels of emotional distress (*p*<.001) than physicians with ALEX or borderline ALEX. These two latter groups showed a significantly decreased (*p*<.001) ability to adopt the point of view of others (i.e. PT) relative to physicians with no ALEX. The direction of correlation coefficients reveal, not only, that higher rates of alexithymia are associated with decreased CS (*r* = .−25, *p*<.001), EC (*r* = .−08, *p*<.001) and PT (*r* = .−25, *p*<.001), but also with increased BO (*r* = .19, *p*<.001), STS (*r* = .39, *p*<.001) and PD (*r* = .30, *p*<.001).

#### Altruism

In order to analyze the way in which altruism influences professional quality of life and empathy, initially correlational analyses were performed on the different domains of ProQoL and IRI and the total score on the helping behavior inventory. Higher rates of prosocial behavior correlated positively and significantly with positive (CS: *r* = .19, *p*<.001) and, although very slightly, with negative (BO: *r* = .07, *p*<.001; STS: *r* = .02, *p*<.001) aspects of medical practice. Positive, significant correlations were also found between altruistic behavior and EC (*r* = .17, *p*<.001) and PT (*r* = .17, *p*<.001), but the association with PD was, as expected, inversely proportional (*r* = −.12, *p*<.001).

**Figure 5:**
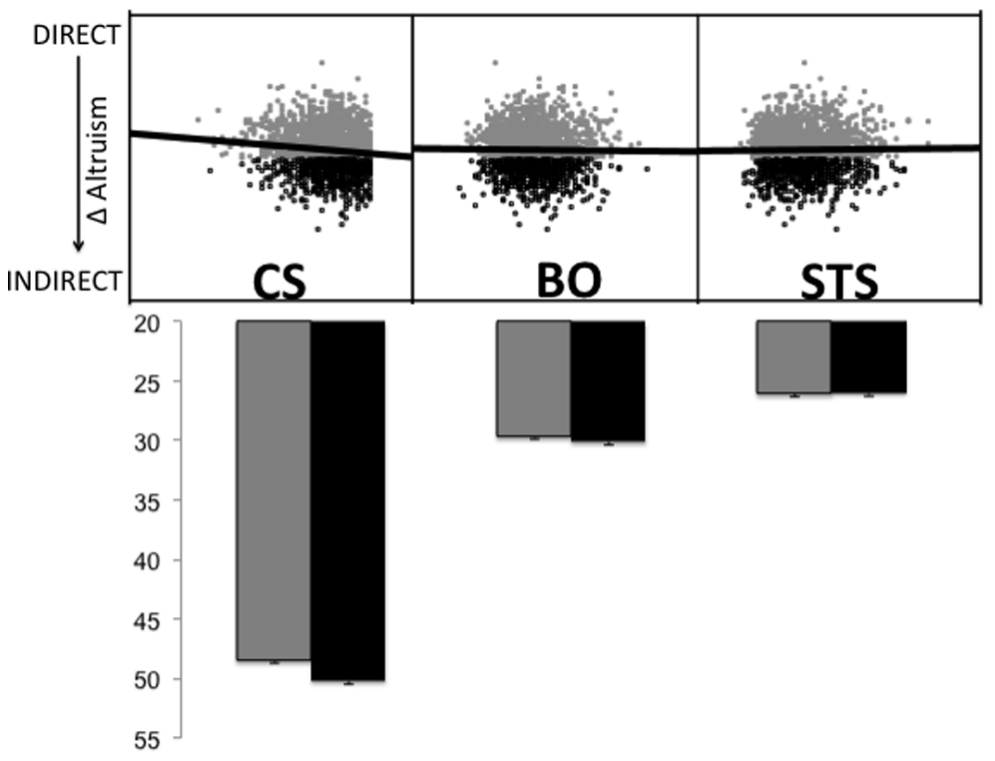
The top of the figure shows correlations between the tendency to favor indirect altruistic acts and the different aspects of professional quality of life. Favoring indirect, abstract, helping behaviors was associated with higher scores of compassion satisfaction. The bar graphs compare scores on Compassion Satisfaction, Burnout, and Secondary Traumatic Stress between physicians who favor direct (ALT-D>ALT-I) and indirect (ALT-I>ALT-D) helping behaviors. Error bars are SEM. Grey dots/bars represent preferably direct helpers, while black bars represent preferably indirect helpers.

Clinical practice dispositions between physicians who were more prone to engaging in direct altruistic behaviors (their ALT-D scores were greater than their ALT-I scores) and those who favored indirect prosocial acts (their ALT-I scores were greater than their ALT-D scores) were then compared. As show in in Figure 5, physicians who tend to favor indirect prosocial acts had higher CS scores (*t*_1879_ = 5.2, *p*<.001, Cohen’s *d* = .24). A significant correlation was also found between the tendency to adopt indirect altruism (ALT-D minus ALT-I scores) and higher scores of CS (*r* = −.12, *p*<.001).

#### Summary

**Figure 6:**
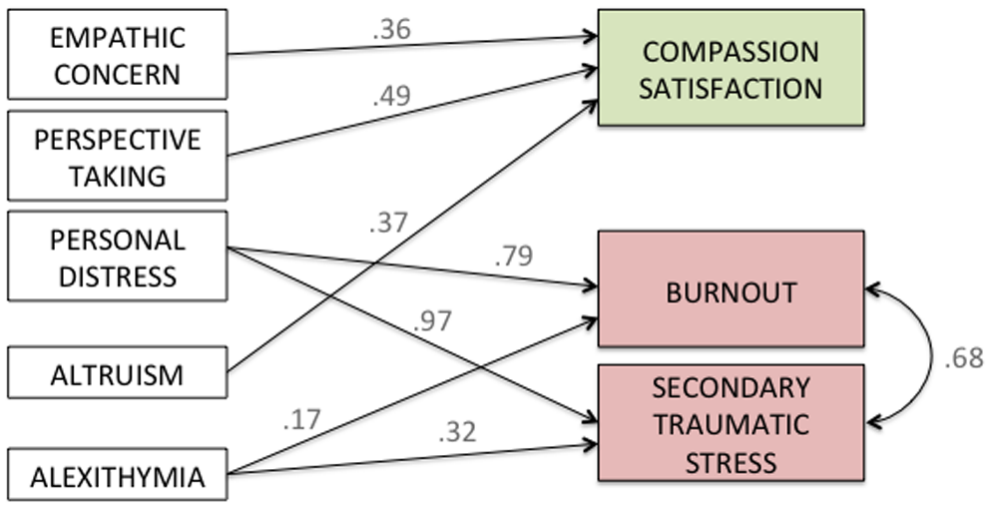
Summary findings from the present study highlighting the positive associations between individual dispositions and the positive and negative aspects of professional quality of life. Values shown next to each arrow represent beta coefficients derived from simple linear regression analyses (i.e. each individual disposition’s effect on the corresponding ProQoL domain).

Figure 6 recapitulates the main positive associations between individual dispositions and aspects of professional quality of life found in the present study in terms of *beta* coefficients derived from simple linear regression analyses (i.e. each individual disposition’s effect on the corresponding ProQoL domain based on the findings above). Overall, empathic concern, perspective taking, and altruism were positively associated with compassion satisfaction. Instead, the negative aspects of professional life, i.e. burnout and secondary traumatic stress, were positively associated with personal distress and altruism.

## Discussion

Despite the well-recognized critical importance of empathy in clinical and care-giving settings for both patients and medical practitioners, a number of studies suggest that practicing physicians may experience difficulties with intersubjective transactions with their patients and that empathy declines during residency training [2], [24]. The contributing factors for such empathy reduction remain murky and are likely to be complex and multifaceted. Therefore, empirical studies with physicians are needed to identify these factors, which individuals are most vulnerable to empathy reduction, and associated maladaptive outcomes such as compassion fatigue and burnout.

The current study investigated the way age, years of experience as a medical practitioner, and individual dispositions, including empathy, personal distress, alexithymia, and altruistic behavior, moderate compassion satisfaction and compassion fatigue in a large number of practicing physicians. These opposite processes result from helping others and are intrinsic properties of physicians’ professional quality of life. This study focused on three aspects in particular: compassion satisfaction, burnout, and secondary traumatic stress following Stamm’s [20] model and widely used questionnaire. Remarkably, the mean scores and standard deviations for each of these three domains matched almost exactly those reported in the original manual, underscoring the strong validity of our large sample.

### Professional Well-being and its Relationship with Sociodemographic Profile

Our data suggest that male and female physicians were comparable in most aspects of their individual dispositions. Their ratings of prosocial behavior and their scores on questionnaires assessing alexithymia and professional quality of life were quite similar, especially after controlling for age and years of experience. This aligns with previous literature, which reports scores on the compassion satisfaction and fatigue domains in the ProQoL questionnaire to be similar between men and women [20].

Previous research has produced inconsistent results regarding the issue of gender differences in professional quality of life. A recent meta-analysis by Purvanova and Muros [25], which looked at the data from 183 studies, found that rather than exhibiting differences in compassion satisfaction and fatigue, women were more emotionally exhausted than men. Empathy scores found in the present study seem to be in line with this account. While men and women physicians were similar in terms of reported personal distress and perspective taking, women displayed significantly higher levels of empathic concern than men. This pattern is consistent with previous studies of gender differences on empathy using the IRI questionnaire [26]–[28] and possible evolutionary roots of empathic concern in women [10]. One possibility, thus, is that increased values of empathic concern among women may come with a cost: emotional exhaustion. This, in turn, has the potential to translate into different everyday work experiences of men and women, as the latter report to feel less valued by patients and their caregivers, as well as by their superiors and colleagues. Interestingly, women also claimed more often than men that their work had affected their personal lives negatively.

No impact of the years of experience working as a physician on either the positive or negative aspects of professional quality of life was observed, especially after controlling for the effect of age. This is also consistent with previous reports documenting that burnout and secondary traumatic stress are similar when comparing participants based on number of years in clinical practice, but that older participants in medical and other working populations are more immune to compassion fatigue [20], [29]–[31].

### Professional Well-being and Individual Dispositions

Understanding the way individual dispositions are related to each other is important, as it may allow for better assessment of factors influencing compassion satisfaction and fatigue. To assess this, multiple regression and principal component analyses were run, revealing that empathic concern and perspective taking, and altruistic behavior, were closely associated with compassion satisfaction. This indicates that the capacities to elicit an other-oriented motivational state for someone in need or distress, while understanding that those feelings are different from one’s own and to exhibit prosocial behavior are good, as they lead to benefits that come from helping patients. This is an important finding, which can be related to the bevy of work which suggests that helping behaviors buffer the effect of exposure to stressful live events [32]. The neuropeptide oxytocin, which stimulates helping behavior, is known to diminish fear and anxiety and decreases activity in the HPA axis as well as blood pressure and cortisol [33]. This may explain why helping behaviors have been demonstrated to be associated with better health and longevity [34].

On the other hand, alexithymia, a difficulty to describe or identify emotions and the tendency to ignore one’s own emotions, as well as personal distress, the set of self-oriented negative emotions resulting from witnessing a distressed person, were associated with both aspects of compassion fatigue, i.e., burnout and secondary traumatic stress. Because different aspects of empathy were more differentially associated with positive and negative aspects of professional life, we investigated how empathy may modulate these aspects. Empathy towards others and the ability to infer others’ thoughts and feelings while understanding that they may differ from an individual’s own was characteristic of participants who experienced the highest levels of compassion satisfaction. These aspects of empathy accuracy, empathic concern and perspective taking can therefore lead to the warm feelings that come from helping those in distress. But when levels of perspective taking are low and accompanied by high levels of personal distress, burnout and secondary traumatic stress emerge. In other words, negative self-oriented emotions elicited by someone in pain/distress with a diminished ability to *take perspective* can lead to compassion fatigue. This is in accordance with literature that has consistently shown that emotional sharing with poor self-regulation can lead to personal distress, which decreases empathic concern and pro-social behavior [35], [36].

### What Contributes to the Down-regulation of Empathy Among Physicians?

Recent affective neuroscience research on physicians casts some light on the role of cognitive regulation or appraisal in medical settings. Two functional neuroimaging studies have directly investigated empathic arousal in physicians in response to the perception of others’ pain. One study used functional MRI to compare the neuro-hemodynamic response in a group of physicians and a group of non-physicians matched controls while they viewed short video clips depicting hands and feet being pricked by a needle (painful situations) or being touched by a cotton bud (non painful situations) [37]. The results demonstrated activation of the regions that belong to the pain matrix, including the anterior cingulate cortex, anterior insula, periaqueductal gray, and somatosensory cortex in the controls when they attended to the painful situations relative to the non-painful ones. This result is in line with previous neuroimaging studies that have demonstrated that the network of regions involved in the experience of physical pain is reliably activated by observing or even imagining another individual in physical or emotional pain [38]. A different pattern of signal change was detected in physicians when they viewed the painful procedures. Cortical regions underpinning executive functions and self-regulation (dorsolateral and medial prefrontal cortex) and executive attention (precentral, superior parietal and temporo-parietal junction) were activated, and unlike in the control group, no signal change was detected in the regions of the pain matrix such as the anterior insula and anterior cingulate cortex. In order to obtain a more precise time-resolution of the information processing during the perception of others in pain, a second study measured event-related potentials (ERPs) from physicians and matched controls as they were presented with the same visual stimuli as in the previous experiment [39]. The results showed an early N110 differentiation between pain and no pain, reflecting negative arousal, over the frontal cortex, as well as a late P300 response over the centro-parietal regions in the control participants. In contrast, no such an early ERP response was detected in the physicians. Altogether, these two studies seem to indicate that the physicians tested have become successful at regulating negative affective arousal. Physicians’ down-regulation of empathic arousal also dampens feelings of unpleasantness that arise from perceiving others’ pain and may have beneficial consequences in freeing up cognitive resources necessary for clinical problem solving. Interestingly, down-regulation of empathy as revealed by these studies may nonetheless imply the enhancement of certain empathic abilities, most likely, empathic concern, and potentially, perspective taking, both of which we found to be associated with compassion satisfaction. This can be evidenced by the higher scores of empathic concern shown by more experienced physicians. The implications of this finding are at least twofold: on the one hand, it is a reminder that the term “empathy” refers to a complex multi-domain complex which must be measured from different angles and approaches; on the other, it is indicative that down-regulation mechanisms of empathy may involve decreasing certain aspects of empathy (e.g. personal distress) while enhancing others (e.g. empathic concern). Future studies that investigate the way emotion regulation, disgust sensitivity, and other affective-related processes influence on empathic regulation will contribute to our understanding of this interesting phenomenon.

In order to further clarify the way in which empathy may modulate compassion satisfaction and fatigue, we studied the extreme groups in our large sample, focusing on those participants who exclusively experience either satisfaction or fatigue from helping others, those who experience both aspects, and those who experience neither. As expected, this latter group showed the lowest values of empathy across all domains relative to the other groups. Physicians who are not only immune from compassion fatigue but who also fail to experience satisfaction from helping others may be over-extending their empathic regulation. They elicit diminished other- and self-oriented emotions and show decreased perspective taking. This stresses the importance of certain minimum levels of empathy, given that by not having them, these physicians are losing the positive outcome of helping their patients, namely, compassion satisfaction [2], [40].

Relative to the group of physicians who suffered burnout and/or secondary traumatic stress with no or very little compassion satisfaction had, again, lower empathic concern and perspective taking, but remarkably higher personal distress.

Because alexithymia was associated with compassion fatigue, it was important to investigate how empathy and professional quality of life were modulated by individual dispositions falling under the umbrella of alexithymia. The rates of alexithymia found in our sample match those reported by other authors using large samples of participants from the general population [e.g., 10% among 1,859 German participants [41]; 10.3% among 2,018 Finnish participants [42]; 9.4% among 5,028 Finnish participants [43]]. As expected from the discussion above, physicians with alexithymia had lower scores on empathic concern and perspective taking and higher scores of personal distress. Accordingly, their inability to identify and describe feelings and their tendency to focus their attention externally, rather than on their own feelings, led to increased rates of burnout and secondary traumatic stress in the absence of compassion satisfaction.

Helping others, eliciting emotions towards them and trying to understand their feelings seem to be beneficiary, as they all lead to compassion satisfaction. In light of this, how is it that helping others may also lead to severe negative consequences? This question was addressed by examining different types of helping behavior. In everyday life, one may engage in altruistic acts in one of two forms: either by helping someone specific, directly (e.g., helping someone carry their shopping bags) or by helping in a more abstract, indirect way (e.g., donating blood). Participants were classified based on the kind of helping behavior that prevailed in each of their lives individually into those who favor direct altruism and those who favor indirect altruism. The latter group had higher scores in compassion satisfaction relative to direct helpers. What is it about helping an abstract entity, an indirect prosocial act, that may lead to more satisfaction? One possibility is that the physician is still helping, i.e., being prosocial, but by doing so in an indirect way, he/she needs not be exposed to the suffering of others. As such, helping becomes more satisfactory. Direct altruism, while still a good thing, may more easily bring about the costs of being compassionate and therefore take away the positive aspects of helping others. Medical clinical practice usually involves helping others directly, making it crucial that strategies be implemented in ways to avoid the costs of being empathic [3].

Finally, another potential contributor to empathy reduction may be that physicians lack the cognitive and emotional resources to engage in empathic processing. Empathy in clinical settings is an effortful exercise that requires cognitive flexibility and high levels of self-regulation [9]. Empirical studies have shown that people have limited capacity for self-regulation and that depletion of cognitive resources reduces empathic concern [44]. These cognitive resources may be particularly restricted, or become limited in physicians due to their demanding and stressful work schedule. Our data support this view, since the group of physicians who experienced very low compassion satisfaction but very high compassion fatigue (CS−CF+) included the highest number of doctors working “on call” shifts, which sometimes last between 36 and 48 hs. On the contrary, physicians who experienced compassion satisfaction in the absence of compassion fatigue (CS+CF−) had the least proportion of participants working this modality. Moreover, the highest number of sick days were taken by CS−CF+ participants, 68.1% of whom had taken a leave of absence due to medical reasons at some point during the last year. Taken together, these results may indicate that overly demanding clinical duties may increase vulnerability for burnout and secondary traumatic stress.

Naturally, more studies are needed with large samples of participants from all over the world in order to determine the generalizability of the associations found with professional wellbeing in the present investigation. If country-, region-, or culture-specific differences are indeed found, it will be important to distinguish whether they result from the diversity of idiosyncratic medical practices around the globe, or else, from the different characteristics of healthcare systems specific to each country/region. Future studies should also explore the way in which individual dispositions and professional well being relate to real-life behavioral measures, such as physicians’ ability to perceive and respond with care to their patient’s needs, as well as patients’ satisfaction. Importantly, the association between professional quality of life and real-life behavioral measures must be studied in other professional categories. This includes health-related fields (e.g. therapists, nurses, etc.), as well as non-health related fields featuring helping professions (e.g. teachers) and non-helping professions (e.g. company lawyers). Exploring what contributes to the various aspects of quality of life across professions with these different occupational profiles can help better understand the specificity or generalizability of the present results, thus allowing for better-tailored empathy training programs aimed at improving professional wellbeing.

### Conclusion

Empathy is an essential element in the physician-patient relationship and a critical aspect of the art of healing. A better understanding of both dispositional and situational factors that mediate empathy and empathy reduction in medical practitioners could point toward effective educational strategies and adaptive coping mechanisms to maintain well-being and patient satisfaction. It is possible that physicians who are most vulnerable to emotional distress and compassion fatigue, which may lead to emotional exhaustion, detachment, and a low sense of accomplishment, are those who have difficulties regulating their negative arousal. It is important to note that a modicum of negative arousal is necessary to help physicians attune to and empathically understand patient’s emotions. The ability to engage in self-other awareness and to regulate one’s emotions is pivotal to the adaptive experience of empathy in clinical practice. These issues require further investigation, including the identification of bio-markers, as the medical profession is struggling to achieve an appropriate balance between clinical distance and empathic concern.

## Supporting Information

Table S1 **Comparison of participant samples based on the set of questionnaires they completed.** Because alexithymia and altruism questionnaires were made available at a later stage of the response period, 5703 physicians responded to the original set of measures and 1881 responded to all measures. We hereby present a comparison of the key variables between these two sub-samples in order to ensure their equivalency in regards to the core variables of the present study.
(DOC)

Table S2 **Comparison of participant samples based on country of residency.** Because the health portal is based in Argentina, and given that this country leads Internet access rankings in Latin America, it was only natural to encounter a bias in our sample of physicians residing in Argentina. 47% of responses came from 22 other countries in Latin America. We hereby present a comparison of the key variables between physicians residing in vs. outside in order to ensure their equivalency in regards to the core variables of the present study.
(DOC)

Table S3 **Comparison of participants based on years of experience.** Because we dichotomized professional experience into “more” vs. “less” experienced physicians based on whether they were above or below the sample’s average years of experience, it was important to test whether other formal ways of dichotomizing this variable held the findings obtained for this comparison. For this reason, we compared empathy and professional quality of life between (A) physicians in percentile 25 vs. percentile 75; and (B) physicians in percentile 10 vs. percentile 9.
(DOC)
