# Supplementary material for: Empathy in Clinical Practice: How Individual Dispositions, Gender, and Experience Moderate Empathic Concern, Burnout, and Emotional Distress in Physicians
Source: PLoS One. 2013 Apr 19;8(4):e61526. doi: 10.1371/journal.pone.0061526 (PMC3631218; doi:10.1371/journal.pone.0061526)
Supplement: Table S1 — Comparison of participant samples based on the set of questionnaires they completed. Because alexithymia and altruism questionnaires were made available at a later stage of the response period, 5703 physicians responded to the original set of measures and 1881 responded to all measures. We hereby present a comparison of the key variables between these two sub-samples in order to ensure their equivalency in regards to the core variables of the present study. (DOC) [file pone.0061526.s001.doc]

|  | Sub-sample of  5703 physicians |  | Sub-sample of  1881 physicians |  | | Statistical comparison |
| --- | --- | --- | --- | --- | --- | --- |
| Age (years) | 45.8 (12.0) |  | 46.7 (11.8) |  | *t* = 2.83, Cohen’s *d* = .06 | |
| Sex (% male) | 56.3% |  | 50.3% |  | | Χ^2^ = 24.3, Cramer’s V = .06 |
| Years of experience | 17.5 (11.7) |  | 18.5 (12.0) |  | | *t* = 2.56, Cohen’s *d* = .06 |
| Empathic Concern | 31.4 (5.2) |  | 31.3 (5.1) |  | | *t* = 0.73, Cohen’s *d* = .02 |
| Personal Distress | 13.1 (4.5) |  | 13.0 (4.5) |  | | *t* = 0.84, Cohen’s *d* = .02 |
| Perspective Taking | 23.7 (4.8) |  | 24.0 (4.8) |  | | *t* = 2.35, Cohen’s *d* = .06 |
| Compassion Satisfaction | 49.8 (9.4) |  | 49.7 (10.0) |  | | *t* = 0.39, Cohen’s *d* = .01 |
| Burnout | 50.1 (9.5) |  | 50.0 (10.0) |  | | *t* = 0.39, Cohen’s *d* = .01 |
| Secondary Traumatic Stress | 49.8 (9.4) |  | 50.2 (10.0) |  | | *t* = 1.57, Cohen’s *d* = .04 |
