# Supplementary material for: Empathy in Clinical Practice: How Individual Dispositions, Gender, and Experience Moderate Empathic Concern, Burnout, and Emotional Distress in Physicians
Source: PLoS One. 2013 Apr 19;8(4):e61526. doi: 10.1371/journal.pone.0061526 (PMC3631218; doi:10.1371/journal.pone.0061526)
Supplement: Table S2 — Comparison of participant samples based on country of residency. Because the health portal is based in Argentina, and given that this country leads Internet access rankings in Latin America, it was only natural to encounter a bias in our sample of physicians residing in Argentina. 47% of responses came from 22 other countries in Latin America. We hereby present a comparison of the key variables between physicians residing in vs. outside in order to ensure their equivalency in regards to the core variables of the present study. (DOC) [file pone.0061526.s002.doc]

|  | Residents of Argentina |  | Residents outside Argentina |  | | Statistical comparison |
| --- | --- | --- | --- | --- | --- | --- |
| Age (years) | 44.4 (12.3) |  | 43.8 (11.7) |  | *t* = 2.16, Cohen’s *d* = .05 | |
| Sex (% male) | 55.9% |  | 54.3% |  | | Χ^2^ = 79.0, Cramer’s V = .10 |
| Years of experience | 18.6 (12.6) |  | 17.8 (11.0) |  | | *t* = 2.56, Cohen’s *d* = .07 |
| Empathic Concern | 31.2 (5.1) |  | 30.7 (5.3) |  | | *t* = 4.18, Cohen’s *d* = .09 |
| Personal Distress | 13.2 (4.5) |  | 12.7 (4.5) |  | | *t* = 4.27, Cohen’s *d* = .09 |
| Perspective Taking | 23.4 (4.9) |  | 23.8 (4.7) |  | | *t* = 4.04, Cohen’s *d* = .09 |
| Compassion Satisfaction | 48.9 (10.2) |  | 50.4 (9.2) |  | | *t* = 6.69, Cohen’s *d* = .15 |
| Burnout | 50.4 (9.9) |  | 49.0 (10.0) |  | | *t* = 6.12, Cohen’s *d* = .14 |
| Secondary Traumatic Stress | 50.3 (9.9) |  | 49.0 (10.0) |  | | *t* = 5.68, Cohen’s *d* = .13 |
