# Supplementary material for: Empathy in Clinical Practice: How Individual Dispositions, Gender, and Experience Moderate Empathic Concern, Burnout, and Emotional Distress in Physicians
Source: PLoS One. 2013 Apr 19;8(4):e61526. doi: 10.1371/journal.pone.0061526 (PMC3631218; doi:10.1371/journal.pone.0061526)
Supplement: Table S3 — Comparison of participants based on years of experience. Because we dichotomized professional experience into “more” vs. “less” experienced physicians based on whether they were above or below the sample’s average years of experience, it was important to test whether other formal ways of dichotomizing this variable held the findings obtained for this comparison. For this reason, we compared empathy and professional quality of life between (A) physicians in percentile 25 vs. percentile 75; and (B) physicians in percentile 10 vs. percentile 9. (DOC) [file pone.0061526.s003.doc]

| (A) | Percentile 25  *n* = 1071 |  | Percentile 75  *n* = 1184 |  | Statistical comparison  *df* = 2253 |
| --- | --- | --- | --- | --- | --- |
| Empathic Concern | 31.0 (5.2) |  | 31.4 (5.1) |  | *t* = 1.98, Cohen’s *d* = .08 |
| Personal Distress | 12.9 (4.4) |  | 13.1 (4.6) |  | *t* = 0.80, Cohen’s *d* = .03 |
| Perspective Taking | 23.5 (4.9) |  | 24.0 (4.7) |  | *t* = 2.17, Cohen’s *d* = .09 |
| Compassion Satisfaction | 48.9 (10.6) |  | 50.1 (9.8) |  | *t* = 2.68, Cohen’s *d* = .11 |
| Burnout | 50.9 (10.0) |  | 49.3 (10.3) |  | *t* = 0.93, Cohen’s *d* = .15 |
| Secondary Traumatic Stress | 50.0 (10.1) |  | 50.5 (9.9) |  | *t* = 1.21, Cohen’s *d* = .04 |

| (B) | Percentile 10  *n* = 906 |  | Percentile 90  *n* = 791 |  | Statistical comparison  *df* = 1695 |
| --- | --- | --- | --- | --- | --- |
| Empathic Concern | 30.7 (5.8) |  | 31.1 (4.8) |  | *t* = 1.18, Cohen’s *d* = .06 |
| Personal Distress | 13.5 (4.7) |  | 13.4 (4.4) |  | *t* = 0.47, Cohen’s *d* = .02 |
| Perspective Taking | 23.4 (5.3) |  | 23.4 (4.7) |  | *t* = 0.44, Cohen’s *d* = .02 |
| Compassion Satisfaction | 48.9 (10.2) |  | 49.2 (9.7) |  | *t* = 0.78, Cohen’s *d* = .04 |
| Burnout | 51.1 (10.1) |  | 49.7 (9.5) |  | *t* = 2.90, Cohen’s *d* = .14 |
| Secondary Traumatic Stress | 50.3 (10.1) |  | 51.5 (9.9) |  | *t* = 2.45, Cohen’s *d* = .12 |
